# Cauda equina compression in an achondroplastic dwarf. Is complex anterior and posterior surgical intervention necessary?

**DOI:** 10.1186/1748-7161-3-18

**Published:** 2008-12-04

**Authors:** George Sapkas, Konstantinos Kateros, Stamatios A Papadakis, Michael Papadakis, George Machairas

**Affiliations:** 1 A' Department of Orthopaedics, University of Athens, Attikon University Hospital, Haidari, Greece; 2 B' Department of Orthopaedics, University of Athens, Agia Olga General Hospital, N. Ionia, Greece; 3 D' Department of Orthopaedics, KAT General Hospital, Kifissia, Greece

## Abstract

We report the case of an achondroplastic dwarf who presented with partial paraplegia due to cauda equina compression. The patient had marked thoracolumbar kyphosis and spinal stenosis at L2–L3. Although only posterior decompression is recommended in the literature for the treatment of achondroplastic patients presenting with neurological problems, a staged anterior and posterior decompression and stabilization was considered necessary for the treatment of this particular patient due to the presence of kyphosis. Satisfactory clinical results were achieved and sustained for six years following this complex operation.

## Background

The incidence of neurologic complications seen in achondroplastic patients varies from 12% to 50% [1] and the first symptoms generally appear in maturity. Posterior decompression with or without posterior stabilization is the usual recommended surgical treatment for the management of such complications [1,2].

We present the clinical case of an achondroplastic patient in whom an extensive, staged anterior and posterior decompression with stabilization was performed, and we stress the need for careful, pre-operative evaluation this type of patients in which spinal stenosis coexists with thoracolumbar kyphosis.

## Case Presentation

A 33-year-old male achondroplastic dwarf was admitted in our Department because of incomplete paraplegia. He had been experiencing low back pain and numbness in both lower extremities, of increasing severity, for the previous 18 months. The patient reported difficulty in walking and in climbing stairs. He also complained of intermittent urinary retention and constipation. On clinical examination, numbness and decreased response to pinprick and light touch below the level of the second lumbar vertebra were present bilaterally. The deep tendon reflexes were decreased and Babinski's sign was negative. Examination of motor function revealed significant bilateral weakness of the hip, ankle and knee flexors and extensors.

**Figure 1:**
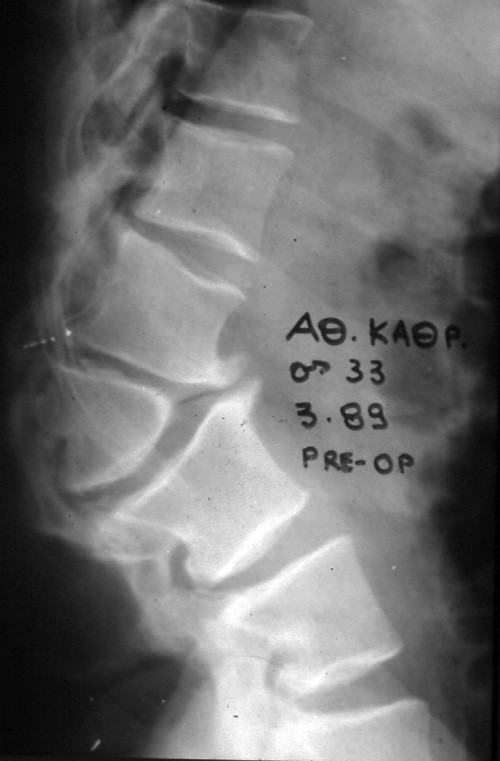
Plain lateral radiograph of patient's lumbar spine showing kyphosis of 70 at the level of the 2^nd ^lumbar vertebral body.

**Figure 2:**
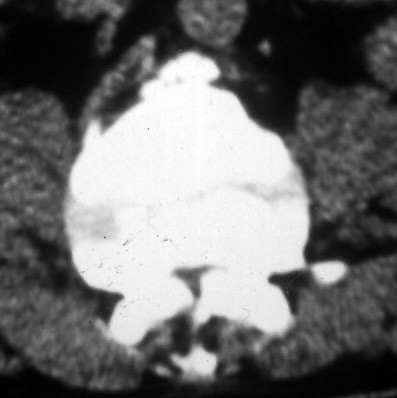
CT-scan of the spine showing severe spinal stenosis.

Plain radiographs revealed marked wedging (kyphosis of 70) of the 2^nd ^lumbar vertebral body (figure 1). On the CT-scan a marked spinal stenosis (T11 to L5) was observed (figure 2) and a myelogram confirmed severe stenosis at L2.

**Figure 3:**
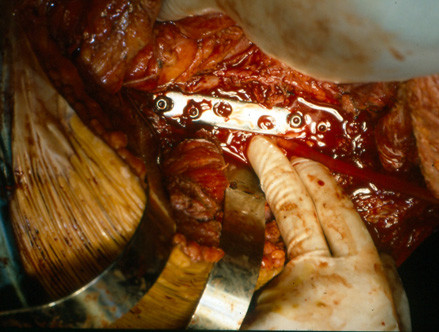
Anterior decompression through retroperitoneal approach. Stabilization with plate and screws, and fusion using iliac autografts (Intra-operative pictures).

The patient underwent an anterior decompression through a retroperitoneal approach. A partial anterior, L1 and L2, corpectomy was performed and following the removal of a sharp protrusion due to disc bulging found at the same level, the cord was considered to be sufficiently decompressed. Stabilization of the spine with a plate and screws, and fusion using iliac autografts comprised the final steps of the operation (figure 3). Following an uneventful post-operative course, the patient was discharged wearing a brace.

The neurological recovery that was observed six months postoperatively was only partial. Therefore a decision was made to further decompress the spine, while in addition a myelogram revealed residual anterolateral cord compression above and below the level of the corpectomy. An extensive posterior laminectomy from the T12 to L5 vertebrae was performed whereupon multiple indentations of the ligamentum flavum were seen at those levels. The neurological condition has markedly improved, and after an extensive follow up period of 12 years, has remained so. The patient was found to be able to walk, having only moderate weakness of the extensors of the right foot, albeit without requiring an ankle support. He has mild residual bilateral paraesthesia over the lateral sides of the femurs, legs and feet and sufficient urinary control.

**Figure 4:**
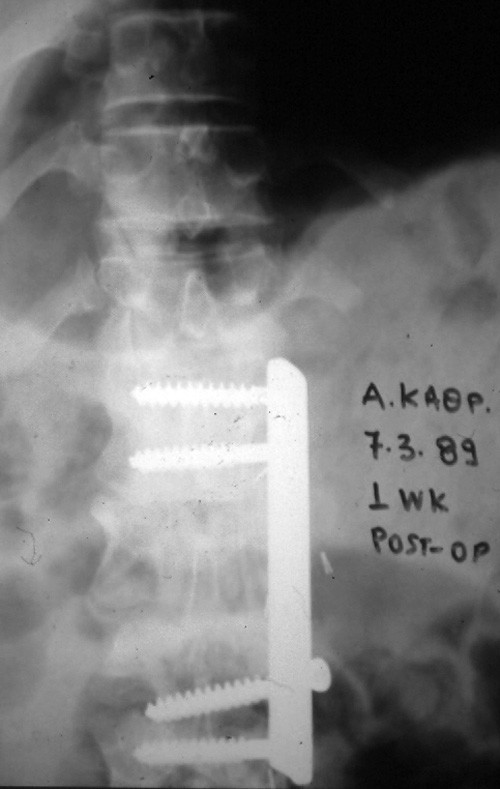
Postoperative anterior-posterior radiograph showing stabilization of the spine with a plate and screws.

**Figure 5:**
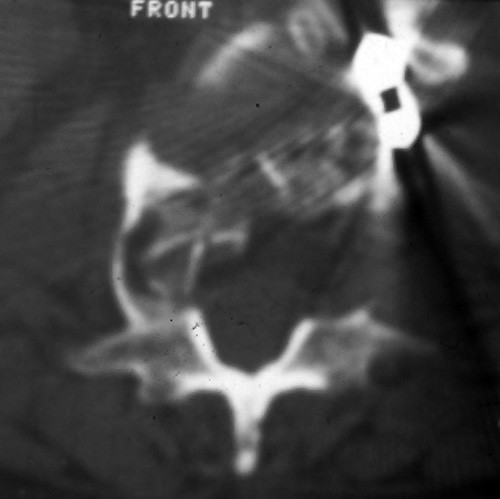
Postoperative CT scan revealed no residual spinal stenosis at the L2 level.

Plain radiographs showed a stable lumbar spine and solid fusion (fig 4). A CT-scan revealed no residual spinal stenosis (fig 5).

## Discussion

Several vertebral anatomical abnormalities have been documented in relation with achondroplasia. [1,3]. Premature synostosis of the ossification centres of the vertebral bodies and the posterior arch cause morphological abnormalities of the spinal column. The laminae become thickened, the pedicles short and stumpy and the vertebral bodies narrow both anteroposterioly and transversely. These abnormalities are more prominent in the lumbar spine. In such an abnormal spine other additional age-related conditions such as disk disease, degenerative changes of the facets and the ligaments and vertebral instability contribute to cause spinal stenosis in adulthood.

Although the existence of lumbar kyphosis in patients with achondroplasia has been described [4], treatment options have has not been adequately emphasized [1,3]. Persistent kyphosis develops in 25–30% of patients with achondroplasia, and 35% of these curves become severe. Such a deformity develops because of wedging or hypoplasia of the vertebral bodies at the thoracolumbar junction. The more severe the kyphosis and the sphenoid malformation of the lumbar vertebrae, the more likely the patient is to develop neurological complications [3,5,6].

Few studies have been carried out in adult patients with achondroplasia with severe thoracolumbar kyphosis and neurological deficits. Accordingly, the optimal procedure for the treatment of kyphosis in adult achondroplastic patients is yet to be established [7].

Posterior surgical decompression by means of laminectomy is the recommended treatment for neurological complications due to spinal stenosis in the general population and is considered effective if performed early [1,2,7-9]. In this particular case, the coexistence of severe lumbar kyphosis and congenital spinal stenosis made the anterior decompression of the lumbar spine absolutely necessary. This was initially considered to be adequate, and it was hoped that any further intervention may not be required. Good results with anterior only decompression have been previously reported in similar patients [3]. Alas, as the patient's improvement would not progress beyond a certain point, posterior decompression had to be performed in addition. With hindsight, the findings of the pre- and post-operative imaging studies, as well as our own intra-operative observations have convinced us that, in cases with marked kyphosis, the only way of relieving cord compression is to perform a staged anterior and posterior decompression with stabilization.

In patients with achondroplasia and neurological defects, the combination of spinal stenosis and thoracolumbar kyphosis has a less favourable prognosis than in those where kyphosis isn't a feature [1]. However, no sufficient explanation has ever been given for this observation.

We strongly suggest that complex deformities in patients with achondroplasia be very carefully evaluated before surgery. Treatment options should be circumspectly deliberated and individualized. It is possible that more complex, staged operations will be necessary, depending on the particular deformity characteristics and patient's features.

## Consent

Written informed consent was obtained from the patient for publication of this case report and any accompanying images. A copy of the written consent is available for review by the Editor-in-Chief of this journal.

## Competing interests

The authors declare that they have no competing interests.

## Authors' contributions

GS, KK, SAP, MP, and GM participated in the design of the study, analysis and writing of this manuscript. SAP, MP and GM participated also in revising critically the manuscript. All authors read and approved the final manuscript.
